# Pyridostigmine as a therapeutic option for pediatric gastrointestinal dysmotilities in ATR-X syndrome. Case report and literature review

**DOI:** 10.3389/fped.2024.1460658

**Published:** 2024-12-17

**Authors:** F. F. Comisi, C. Soddu, M. Corpino, M. Marica, R. Cacace, T. Foiadelli, S. Savasta

**Affiliations:** ^1^ Pediatric Clinic and Rare Diseases, Microcitemico Hospital “A. Cao”, University of Cagliari, Cagliari, Italy; ^2^ Pediatric Clinic and Rare Diseases, Microcitemico Hospital “A. Cao”, Cagliari, Italy; ^3^ Pediatric Clinic, Fondazione IRCCS Policlinico San Matteo, Pavia, Italy

**Keywords:** gastrointestinal dysmotility, alpha thalassemia, alpha-thalassemia X-linked intellectual disability, ATR-X syndrome, pyridostigmine, constipation, therapy

## Abstract

**Background:**

Alpha-thalassemia X-linked intellectual disability (ATR-X) syndrome, is a rare genetic disorder, caused by mutations in the ATRX gene. Clinical manifestations include typical facial dysmorphisms, mild-to-severe intellectual disability, hypotonia, genital anomalies, significant gastrointestinal (GI) complications, such as abdominal distension, chronic constipation, feeding difficulties, gastroesophageal reflux, and mild-to-moderate anemia secondary to alpha-thalassemia.

**Case presentation:**

We report a patient with ATR-X syndrome suffering from gastrointestinal dysmotility and highlight the beneficial effects of pyridostigmine. Knowledge about the role and appropriate dosage of pyridostigmine in GI motility disorders is limited. To date, only nine pediatric cases involving pyridostigmine for GI dysmotility have been reported.

**Conclusions:**

Considering current understanding about the treatment of gastrointestinal complications in patients with genetic syndromes, this case provides new insights into management of these complex clinical presentations.

## 1 Introduction

Alpha-thalassemia X-linked intellectual disability (ATR-X) syndrome (ATR-X, OMIM Entry#301040) is a rare disorder with complex clinical manifestations (1). The first association between alpha-thalassemia and intellectual disability was reported in 1981 by Weatherall and colleagues (2). Since the identification of the ATRX gene, located on Xq13, as causative of the ATR-X syndrome (3), more than 130 families and 200 affected individuals have been described (4) with an estimated incidence of less than 1/100,000 live-born males (5). The hallmarks of the ATR-X syndrome are distinctive craniofacial features, genital anomalies, hypotonia, and mild-to-severe developmental delay/intellectual disability (DD/ID). Craniofacial dysmorphisms include small head circumference, telecanthus or widely spaced eyes, short triangular nose, tented upper lip, and thick or everted lower lip with coarsening of the facial features over time. Genital anomalies range from hypospadias and cryptorchidism to severe hypospadias and ambiguous genitalia. Alpha-thalassemia, observed in about 75% of affected individuals, is mild and typically does not require treatment (6). A risk of early-onset osteosarcoma has been reported in a few males with germline pathogenic variants (6). Gastrointestinal complications, as well as in many genetic syndromes (7, 8), affect most of the patients, significantly contributing to morbidity (6). Recurrent vomiting or regurgitation is a common finding and seems likely to be a manifestation of a more generalised gut dysmotility. Aspiration is a common cause of death in early childhood. Excessive drooling, aerophagia, food refusal, and feeding-associated distress in these children are common. Constipation is also frequent and might be a major issue in some patients (9, 10). Notably, pseudo-volvulus, ultra-short Hirschprung disease and colonic hypoganglionosis have been reported too (9). The primary aim of this paper is to identify new therapeutic strategies for ATR-X syndrome-related GI complications, suggesting pyridostigmine as a viable alternative to the commonly utilized drug classes.

## 2 Case report

**Figure 1:**
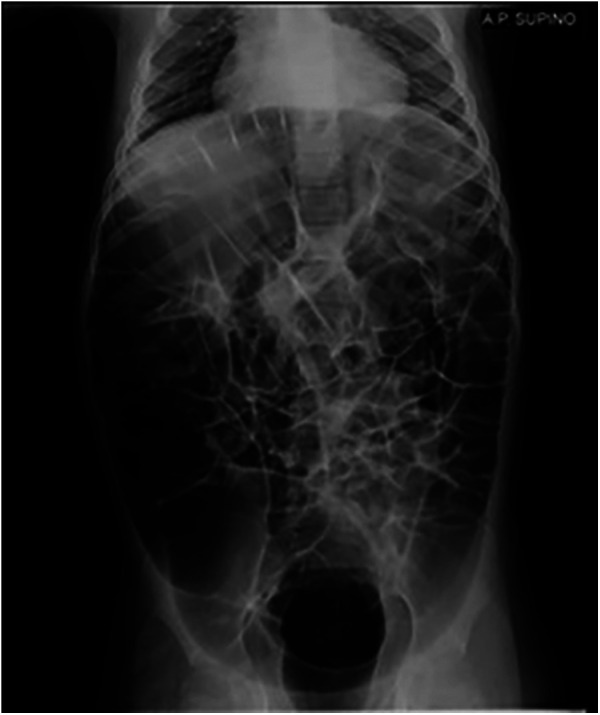
Upright plain abdominal x-ray showing massive dilation of the intestinal loops.

The patient is a 10-year-old caucasian male, born at 39 weeks' gestation following a pregnancy complicated by oligohydramnios, with a spontaneous vaginal delivery. His parents are non-consanguineous, and the family pedigree revealed no significant genetic history. Hypotonia was noted during the perinatal period. At one year of age, molecular analysis of the ATRX gene identified a pathogenic hemizygous *c.736C* *>* *T* variant. His clinical phenotype includes characteristic facial dysmorphisms (microcephaly, low-set hairline, telecanthus, a small triangular nose with anteverted nostrils, and a carp-shaped mouth with full lips), psychomotor delay, epilepsy, moderate bilateral hearing impairment, and laryngomalacia without impact on respiratory function. He was admitted to our department with significant gastrointestinal issues, including chronic gastroesophageal reflux (GER), dysphagia, and severe, persistent constipation. He had recently been hospitalized three times due to the worsening of his GI symptoms. Abdominal x-ray revealed gastroparesis and severe intestinal loop distension (Figure 1). Furthermore, barium enema showed a sub-stenotic region in the rectosigmoid area. Treatment with macrogol and enemas was ineffective. Persistent, severe abdominal pain and distension led to distress and agitation. After cardiac evaluation, he was started on oral pyridostigmine at a dose of 30 mg/day (1.6 mg/kg/day), gradually increased to 60 mg/day (3.2 mg/kg/day) within 20 days. This approach avoided the need for surgery, notably improved the patient's bowel habit, and reduced abdominal discomfort, leading to a significant decrease in irritability and an overall enhancement in his quality of life. He continued on a maintenance dose of oral pyridostigmine at 45 mg/day, and after a year of sustained treatment, his gastrointestinal symptoms fully resolved, with no recurrence of abdominal distension, fecal stasis, nighttime awakenings, or significant side effects.

## 3 Discussion

Gastrointestinal manifestations are common findings in ATR-X syndrome, such as feeding problems, vomiting, abdominal distension, and chronic constipation (10). According to Leon and colleagues 30% of patients suffer from GI complications (4). In 2006 Martuciello et al. reviewed 128 cases of ATR-X syndrome patients with GI manifestations, most commonly gastroesophageal reflux (GER), drooling and constipation. Patient's refusal for food is relatively common among these patients and at times leads to dehydration. Intestinal malrotation was a rare finding in Martucciello's review but it was the cause of death in two out of four patients described (10). Watanabe and colleagues investigated the gastroesophageal function of a child with ATR-X syndrome, presenting with reduced esophageal clearance and gastric volvulus. Laparoscopic anterior gastropexy was conducted, and a button PEG-J was inserted, resulting in a better nutritional management and quality of life (11). In 2015 Horesh et al. reported a patient with recurrent large bowel volvulus, who kept on suffering from food refusal, abdominal distension, and dehydration after subtotal colectomy (1). Management of GI motility disorders is challenging, with limited medical and surgical options. Pyridostigmine, an acetylcholinesterase inhibitor (CI), may be considered in such patients when other treatments have not been beneficial. CIs are well known drugs, mainly used in general anesthesia and in the symptomatic treatment of patients with myasthenia gravis (12). In the GI tract, the inhibition of choline acetyl esterase enzyme increases the availability of acetylcholine at the neuro-neuronal synaptic cleft within the enteric nervous system as well as at neuromuscular junctions (13). Little is known about the role and dosage of pyridostigmine in pediatric GI motility disorders. In 2009, Boybeyi and colleagues reported a 3-year-old patient with pediatric intestinal pseudo-obstruction (PIPO), successfully treated with pyridostigmine 30 mg/Kg/day (14). Manini et al. presented a case series of four pediatric patients with abdominal distension and other dysmotilities. Administration of pyridostigmine markedly improved GI symptoms, with 100% success rate and with side effects (abdominal pain and cramps) in one case only (15). Similar results were obtained by Choudury, who reported the case of a 9-year-old girl with a long-term history of constipation and intermittent abdominal distension, since the age of 3 years (16). Different types of laxatives were used, including polyethylene glycol, lactulose, senna, sodium picosulfate and docusate sodium, with no definitive effect. She was started on enteral pyridostigmine at a dose of 0.5 mg/Kg twice per day and gradually increased to 1 mg/kg twice per day. An abdominal x-ray confirmed her clinical improvement. Lee et al. described the use of pyridostigmine in two children with chronic intestinal pseudo-obstruction harbouring ACTG2 gene variants (17). After 10 days of intravenous neostigmine (0.5 mg in 50 ml normal saline) at 0.5 mg per hour, the treatment was switched to oral pyridostigmine at a dose of 180 mg/day (case 1) and 7 mg/kg/day (case 2) without side effects. In response to pyridostigmine treatment, the length of hospital stay and dependency on parenteral nutrition were reduced in both patients (17). In 2019 a 2-year and 4-month- old girl was presented by Di Nardo and colleagues (13). Chronic constipation, food refusal, poor weight and height gain were her main symptoms. Pyridostigmine 2 mg/kg/twice per day, then increased to 3 mg/kg twice per day, led to improvement of gut motility with reduction of abdominal distension, disappearance of vomiting and, occurrence of spontaneous bowel movements (13) (Table 1).

**Table 1:** Published studies on the effects of pyridostigmine in pediatric gastrointestinal dysmotilities. Adapted and updated from Di Nardo et al. 2019 (13).

Authors	Patients *n*	Age	Clinical features	Pyridostigmine dose	Outcome	Side effects
Boybeyi et al. (14)	1	3 y	Neuropathic PIPO presenting with abdominal distension, intolerance to oral feeding, constipation	30 mg/kg/day	Resolution of abdominal distension, improved oral intake, increased bowel movements	None
Manini et al. (15)	4	18 y	Abdominal distension, intolerance to enteral feeds and bilious emesis	0,3 mg/kg/day	Decreased abdominal distension, increased enteral calories, decreased TPN	None
		7 y	Chronic abdominal distension, constipation, feeding intolerance, and vomiting	4 mg/kg/day decreased to 0,75 mg/kg/day	Decrease abdominal distension, increased bowel movement frequency, resolved vomiting with better tube feeding tolerance	None
		11 y	Ileus with abdominal distension, vomiting, abdominal pain, decreased stool output	1,7 mg/kg/day increased to 1,1 mg/kg/day	Immediate improvement in distension, pain and vomiting	Abdominal pain and cramps
		10 y	Abdominal pain, vomiting, abdominal distension	1,1 mg/kg/day increased to 2,2 mg/kg/day	Improvement in vomiting and abdominal distension	None
Choudury et al. (16)	1	9 y	Myopathic PIPO presenting with abdominal distension and pain, vomiting and constipation	0,5 mg/kg twice per day, increased to 1 mg/kg twice per day	Reduction of abdominal distension and gastric drainage, tolerance of enteral feeding	None
Di Nardo et al. (13)	1	2 y 4 m	Constipation, food refusal, abdominal distension	2 mg/kg twice per day, increased to 3 mg/kg twice per day	Disappearance of vomiting, reduction of abdominal distension, occurrence of spontaneous bowel movements	None
Lee et al. (17)	2	11 y	Myopathic PIPO presenting with congenital myotonic dystrophy, small bowel and colonic distension, megacystis	150 mg/day	Reduction in length of hospital stay and need of parenteral nutrition	None
		5 y		7 mg/kg/day		

## 4 Conclusions

ATR-X syndrome is characterized by a wide range of severe gastrointestinal symptoms, including abdominal distension and chronic constipation. The therapeutic potential of pyridostigmine in this context is a significant finding. The present paper adds information to the few experiences currently available in literature. According to our experience and reported cases, pyridostigmine may be a suitable alternative when first-line treatments are ineffective, as they were in our case. Its off-label use seems to be further supported by a low side-effect profile; of the nine patients documented in the literature, only one experienced minor side effects (abdominal pain and cramps), with no adverse effects observed in the remaining cases or in ours. Optimal dosage, long-term efficacy, and side effects of pyridostigmine in pediatric GI motility disorders remain an area for future research. A deeper understanding of GI complications in ATR-X syndrome is the goal to determine the most effective pyridostigmine dosage for these patients.

## Data Availability

The original contributions presented in the study are included in the article/Supplementary Material, further inquiries can be directed to the corresponding author.
